# Haplotype-Specific Expression Analysis of MHC Class II Genes in Healthy Individuals and Rheumatoid Arthritis Patients

**DOI:** 10.3389/fimmu.2021.707217

**Published:** 2021-08-17

**Authors:** Miranda Houtman, Espen Hesselberg, Lars Rönnblom, Lars Klareskog, Vivianne Malmström, Leonid Padyukov

**Affiliations:** ^1^Division of Rheumatology, Department of Medicine, Karolinska Institutet, Karolinska University Hospital, Stockholm, Sweden; ^2^Department of Medical Sciences, Rheumatology, Science for Life Laboratory, Uppsala University, Uppsala, Sweden

**Keywords:** major histocompatibility complex, RNA-sequencing, allelic expression, T cell, monocyte, rheumatoid arthritis

## Abstract

*HLA-DRB1* alleles have been associated with several autoimmune diseases. For anti-citrullinated protein antibody positive rheumatoid arthritis (RA), *HLA-DRB1* shared epitope (SE) alleles are the major genetic risk factors. In order to study the genetic regulation of major histocompatibility complex (MHC) Class II gene expression in immune cells, we investigated transcriptomic profiles of a variety of immune cells from healthy individuals carrying different *HLA-DRB1* alleles. Sequencing libraries from peripheral blood mononuclear cells, CD4+ T cells, CD8+ T cells, and CD14+ monocytes of 32 genetically pre-selected healthy female individuals were generated, sequenced and reads were aligned to the standard reference. For the MHC region, reads were mapped to available MHC reference haplotypes and AltHapAlignR was used to estimate gene expression. Using this method, *HLA-DRB* and *HLA-DQ* were found to be differentially expressed in different immune cells of healthy individuals as well as in whole blood samples of RA patients carrying *HLA-DRB1* SE-positive *versus* SE-negative alleles. In contrast, no genes outside the MHC region were differentially expressed between individuals carrying *HLA-DRB1* SE-positive and SE-negative alleles, thus *HLA-DRB1* SE alleles have a strong cis effect on gene expression. Altogether, our findings suggest that immune effects associated with different allelic forms of HLA-DR and HLA-DQ may be associated not only with differences in the structure of these proteins, but also with differences in their expression levels.

## Introduction

**Figure 1:**
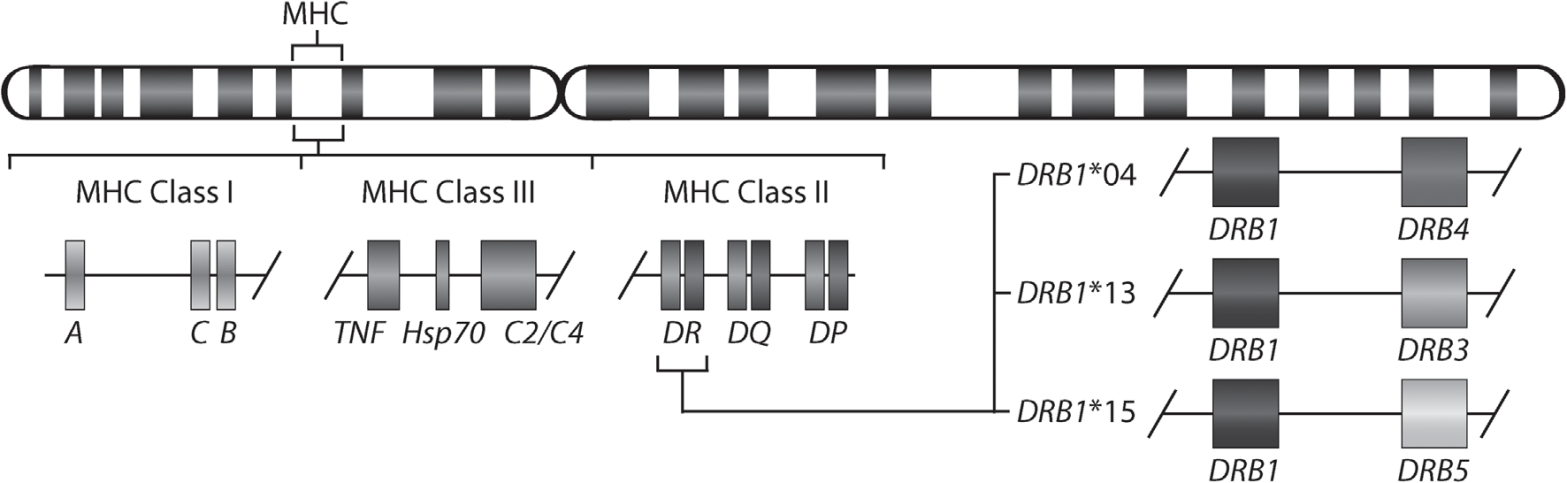
Gene map of the major histocompatibility complex (MHC) region. The MHC region on the short arm of human chromosome 6 contains the HLA-DR (HLA-DRA and HLA-DRB) molecules. The allelic variants of *HLA-DRB1* (DRB1*04, *13, and *15) are linked with only one of the genes *HLA-DRB4*, *HLA-DRB3*, or *HLA-DRB5*.

Rheumatoid arthritis (RA) is a chronic inflammatory disorder affecting approximately 0.5-1% of the population worldwide (1). Although the exact cause of RA remains unknown, a set of so-named shared epitope (SE) alleles, HLA-DRB1*01 (*01:01 and *01:02), *04 (*04:01, *04:04, *04:05, and *04:08), and *10 (*10:01), have been associated with RA (2) and more specifically with anti-citrullinated protein antibody (ACPA)-positive RA (3). These alleles share a sequence encoding five amino acids in position 70-74 of the antigen-binding groove of the HLA-DR beta chain. More recent studies have shown the importance also of amino acids in positions 11 and 13 in the HLA-DR beta chain (4). It has been suggested that citrullinated antigens may bind preferentially to HLA-DRB1 SE sequences leading to the activation of autoreactive T-cells (5). *HLA-DRB1* SE alleles are not the only alleles associated with ACPA-positive RA, a meta-analysis has described that HLA-DRB1*13:01 alleles provide protection against ACPA-positive RA (6). Indeed, many autoimmune diseases are associated with certain *HLA-DRB1* alleles. For example, HLA-DRB1*15:01 confers the strongest risk for developing multiple sclerosis (7), but is not associated with RA. Moreover, the genetic architecture of the HLA locus is complex as allelic variants of *HLA-DRB1* involve linkage with either none or one of the paralogs (*HLA-DRB3*, *HLA-DRB4* or *HLA-DRB5*). Therefore, *HLA-DRB1* is expressed in cells with all *HLA-DRB1* haplotypes, whereas expression of the paralogs is haplotype-specific (**Figure 1**).

The genetic association of *HLA-DRB1* to RA is proposed to at least partly reflect a favored binding of citrullinated peptides to the HLA binding groove. However, the precise molecular mechanisms by which *HLA-DRB1* SE alleles predispose to ACPA-positive RA are currently unclear. There is evidence suggesting that expression of genes in the major histocompatibility complex (MHC) region vary significantly between different *HLA-DRB1* alleles (8–11). In addition, a recent study showed allele-specific expression of *HLA-DRB1* in Korean ACPA-positive RA patients (12). However, these differences have not been studied in healthy individuals with susceptibility alleles in the MHC locus and for specific cell types. In this study, we aimed to identify differentially expressed genes in peripheral blood mononuclear cells (PBMCs) and isolated CD4+ T cells, CD8+ T cells, and CD14+ monocytes from PBMCs of healthy individuals with *HLA-DRB1* SE alleles compared to healthy individuals not carrying these alleles, which will be referred to as SE negative alleles. In addition, the total expression of the MHC Class II molecules in whole blood samples was investigated in both healthy individuals and in RA patients. We used RNA-seq data with a pipeline [AltHapAlignR (8)] that aligns reads to available MHC reference haplotypes for more accurate analysis of gene expression in the MHC region. This analysis will help us to understand the mechanisms of immune response in individuals with susceptibility alleles in the MHC locus.

## Materials and Methods

### Ethics Statement

This project was undertaken with ethical approval of the Regional Ethical Review Board in Uppsala (2009/013) and all healthy donors gave written informed consent according to the declaration of Helsinki.

### Blood Donors

Blood samples from 32 healthy previously genotyped donors (females between 55 and 73 years of age) were provided by the Uppsala Bioresource (Uppsala University Hospital, Uppsala, Sweden). The samples were selected by positivity for certain *HLA-DRB1* alleles (HLA-DRB1*04, HLA-DRB1*13:01, and HLA-DRB1*15:01). The main characteristics of the participants are presented in **Table 1**.

**Table 1:** Characteristics of study participants.

Sample ID	Sex	Age	*HLA-DRB1* alleles	*HLA-DRB1* SE	PBMCs	CD4+ T cells	CD8+ T cells	CD14+ monocytes
1	F	60	*03:01/*04:01	Positive	x	x	x	NA
2	F	58	*03:01/*04:01	Positive	x	NA	NA	NA
3	F	59	*03:01/*15:01	Negative	x	NA	x	NA
4	F	65	*03:01/*15:01	Negative	NA	NA	x	NA
5	F	56	*01:01/*04:01	Double positive	x	x	x	NA
6	F	65	*03:01/*13:01	Negative	x	x	NA	NA
7	F	65	*04:01/*07:01	Positive	x	x	x	NA
8	F	60	*04:01/*13:03	Positive	NA	x	x	NA
9	F	65	*04:01/*07:01	Positive	x	x	NA	x
10	F	59	*03:01/*04:01	Positive	x	x	NA	x
11	F	63	*03:01/*15:01	Negative	x	x	NA	NA
12	F	61	*04:01/*14:01	Positive	x	x	x	NA
13	F	67	*03:01/*13:01	Negative	NA	x	NA	NA
14	F	61	*03:01/*15:01	Negative	x	x	NA	NA
15	F	65	*01:01/*04:01	Double positive	x	x	x	x
16	F	56	*03:01/*04:01	Positive	x	x	x	NA
17	F	62	*03:01/*15:01	Negative	x	x	NA	x
18	F	62	*03:01/*13:01	Negative	x	x	x	NA
19	F	70	*03:01/*04:01	Positive	x	x	NA	NA
20	F	55	*03:01/*13:01	Negative	x	x	x	NA
21	F	73	*03:01/*13:01	Negative	x	x	NA	NA
22	F	59	*04:01/*04:04	Double positive	x	NA	x	NA
23	F	58	*03:01/*13:01	Negative	x	x	x	NA
24	F	66	*04:01/*14:01	Positive	x	NA	NA	x
25	F	61	*04:01/*09:01	Positive	x	x	x	NA
26	F	61	*03:01/*04:01	Positive	x	NA	x	NA
27	F	72	*01:01/*04:01	Double positive	x	x	x	x
28	F	67	*03:01/*13:01	Negative	x	x	x	x
29	F	56	*04:08/*07:01	Positive	x	x	x	NA
30	F	67	*03:01/*15:01	Negative	x	x	x	x
31	F	62	*03:01/*15:01	Negative	x	x	x	x
32	F	57	*01:01/*04:01	Double positive	x	x	x	x

F, female; NA, not available; PBMCs, peripheral blood mononuclear cells; SE, shared epitope; x, included in analyses.

### Blood Sampling and Cell Separation

Buffy coats were processed by density gradient centrifugation using Ficoll (GE Healthcare Bio-Sciences AB, Uppsala, Sweden) and PBMCs were subsequently recovered. CD4+, CD8+, and CD14+ cells were isolated from the PBMCs *via* positive selection using CD4, CD8 or CD14 Microbeads (Miltenyi Biotec Norden AB, Lund, Sweden) on the autoMACS® Pro Separator (Miltenyi Biotec Norden AB).

### RNA Sequencing

Total RNA was extracted with the RNeasy Mini kit (Qiagen AB, Sollentuna, Sweden) according to manufacturer’s instructions. Samples were treated with DNase (Qiagen) for 20 min at room temperature to avoid contamination with genomic DNA. The quality of each RNA sample was assessed using the Agilent Bioanalyzer 2100 and RNA 6000 Nano Chips (Agilent Technologies Sweden AB, Kista, Sweden). The RNA integrity number (RIN) ranged between 3.4 and 9.5 (median of 7.9). The RNA was fragmented and prepared into sequencing libraries using the Illumina TruSeq stranded total RNA sample preparation kit with ribosomal depletion using RiboZero (2 x 125 bp) and sequenced on an Illumina HiSeq 2500 sequencer (SNP&SEQ Technology Platform, Uppsala, Sweden). Between 24.5 and 60.5 (median of 41) million reads were produced per sample. Raw read quality was evaluated using FastQC. Pre-filtering on quality of reads using cutadapt (version 1.9.1) was applied (-q 30 -a AGATCGGAAGAGCACACGTCTGAACTCCAGTCAC -A AGATCGGAAGAGCGTCGTGTAGGGAAAGAGTGTAGATCTCGGTGGTCGCCGTATCATT -m 40). Filtered reads were aligned to the hg38 assembly, containing the MHC reference haplotype PGF [GENCODE release 28 (GRCh38.p12)], using STAR in two-pass mode (version 2.5.4b) (13) with default settings. STAR was also used to obtain the gene counts. All sequencing data generated in this study are available at NCBI Gene Expression Omnibus accession number GSE163605.

### Whole Blood RNA Sequencing Data of Healthy Individuals and RA Patients

FASTQ files for 439 whole blood samples were downloaded from the GTEx project (14). For RA patient samples, 158 FASTQ files from the EIRA/RECOMBINE project were used. Seq2HLA (15) was used to impute classical HLA alleles from RNA-seq data. Individuals with HLA-DRB1*03, *04, *07 and *15 alleles were selected (based on the available MHC reference haplotypes APD, COX, DBB, MANN, MCF, PGF, QBL and SSTO). Mapping to the human genome, MHC region and differential gene expression analysis were performed as described above.

### Mapping to the MHC Region

Counts of genes in the MHC region (chr6: 28,500,000-33,500,000) were replaced with counts obtained from AltHapAlignR (8). In short, unmapped reads and reads mapped to the MHC region (using MHC reference haplotype PGF) were extracted and realigned to all available MHC reference haplotypes (APD, COX, DBB, MANN, MCF, QBL, SSTO) independently using STAR in two-pass mode. Reads mapped to multiple regions, with mapping quality less than 20, and duplicate reads were removed before further analyses.

### Differential Gene Expression Analysis

Raw expression counts obtained from STAR (non-MHC genes) and AltHapAlignR (MHC genes) were adjusted for library size using the R package DESeq2 (version 1.26.0) (16). Pre-filtering of low count genes was performed to keep only genes that had at least 50 counts in total over all samples. The counts were regularized-logarithm transformed for principal component analysis (PCA). For each specific cell subset, RIN score was highly correlated with principal component 1 and used as covariate as binary category (< 7 and ≥ 7) in the analyses. The default DESeq2 options were used, including log fold change shrinkage using apeglm (17) and independent hypothesis weighting (18). *P*-values were obtained with the Wald test. Differences in gene expression with Benjamini-Hochberg adjusted *P*-value < 0.05 and fold change (log2) > 1 were considered significant.

### HLA-Typing and Imputation

For the 32 healthy donors, HLA-DRB1 genotypes were initially imputed from Immunochip data by SNP2HLA (19) and later HLA low resolution typing was performed for validation. Additionally, DR4 subtyping was performed for HLA-DRB1*04 positive individuals. *HLA*-typing was performed by sequence-specific primer polymerase chain reaction assay (DR low-resolution kit and DR4 kit; Olerup SSP, Stockholm, Sweden) and analyzed by agarose gel electrophoresis (20). An interpretation table was used to determine the specific genotype according to the manufacturer’s instructions. In addition, seq2HLA (15) was used to impute classical HLA alleles from RNA-seq data for all cohorts.

### Quantitative Real-Time PCR

RNA of the PBMC, CD4+, CD8+, and CD14+ cell subset samples was converted into cDNA using iScript™ Reverse Transcription Supermix (Bio-Rad, Solna, Sweden). Quantitative real-time PCR (qPCR) was carried out using SsoAdvanced™ Universal SYBR Green Supermix (Bio-Rad) and primers detecting *HLA-DQA* (forward primer 5’-CAACATCACATGGCTGAGCA-3’ and reverse primer 5’-TGCTCCACCTTGCAGTCATAA-3’), *HLA-DQB* (forward primer 5’-TCTCCCCATCCAGGACAGAG-3’ and reverse primer 5’-TTCCGAAACCACCGGACTTT-3’), and *HLA-DRA* (forward primer 5’-CCTGTCACCACAGGAGTGTC-3’ and reverse primer 5’-TCCACCCTGCAGTCGTAAAC-3’) on a CFX384 Touch™ system (Bio-Rad) with the following protocol: 95°C for 30 sec, followed by 40 cycles of 95°C for 10 sec and 60°C for 30 sec. The endogenous controls *ACTIN* (forward primer 5’-GGACTTCGAGCAAGAGATGG-3’ and reverse primer 5’-AGCACTGTGTTGGCGTACAG-3’), *UBE2D2* (forward primer 5’-TGCCTGAGATTGCTCGGATCT-3’ and reverse primer 5’-TCGCATACTTCTGAGTCCATTCC-3’), and *ZNF592* (forward primer 5’-GTAAAGGAGAATTGCCTGCA-3’ and reverse primer 5’-GAATGCACATTTGTGGAAAA-3’). Data was analyzed with the SDS 2.4 software of Applied Biosystems before applying the ΔΔCt method (21).

### Cell Type Enrichment Analysis

The xCell tool (22) was used to estimate cellular heterogeneity in the PBMC, CD4+, CD8+, and CD14+ cell subsets from RNA sequencing data. xCell uses the expression levels ranking [Transcripts Per Million (TPM)] and these were obtained using Salmon (version 0.8.2) (23).

## Results

### Differentially Expressed Genes in PBMCs of *HLA-DRB1* SE-Positive *Versus* SE-Negative Healthy Individuals

**Figure 2:**
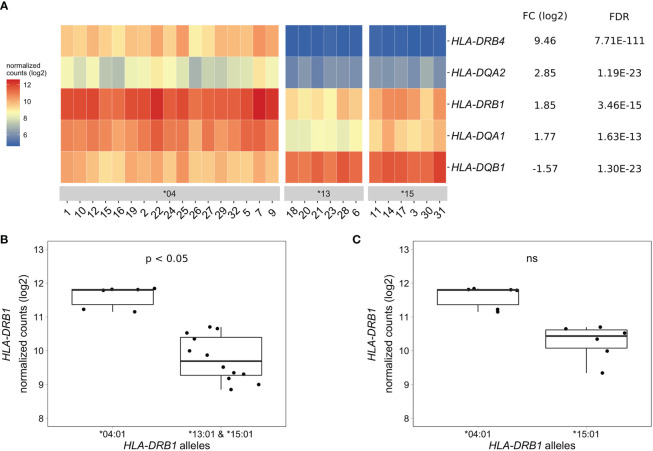
Differentially expressed MHC Class II genes in PBMCs of *HLA-DRB1* SE-positive *versus* SE-negative healthy individuals. **(A)** Heat map of differentially expressed genes (log transformed normalized gene counts) in PBMCs of *HLA-DRB1* SE-positive [*04 (*n* = 17)] *versus* SE-negative [*03:01/*13:01 and *03:01/*15:01 (*n* = 12)] individuals. Genes shown in red have higher gene counts and those shown in blue have lower gene counts. **(B)** *HLA-DRB1* gene expression in PBMCs of *HLA-DRB1* SE-positive [*03:01/*04:01 (*n* = 6)] and SE-negative [*03:01/*13:01 and *03:01/*15:01 (*n* = 12)] individuals. **(C)** *HLA-DRB1* gene expression in PBMCs of *HLA-DRB1* SE-positive [*03:01/*04:01 (*n* = 6)] and SE-negative [*03:01/*15:01 (*n* = 6)] individuals. FC, fold change; ns, non-significant adjusted *P*-value (FDR).

To identify differentially expressed genes in PBMCs of healthy individuals with and without *HLA-DRB1* SE alleles, we conducted RNA-seq on 29 PBMC samples and aligned reads to available MHC reference haplotypes using AltHapAlignR (8). We identified five MHC Class II genes (*HLA-DRB4*, *HLA-DQA2*, *HLA-DRB1*, *HLA-DQA1*, and *HLA-DQB1*) that were differentially expressed to a great degree in PBMCs of *HLA-DRB1* SE-positive *versus* SE-negative individuals (**Figure 2A** and **Table S1**). Of these differentially expressed genes, *HLA-DRB4*, *HLA-DQA2*, *HLA-DRB1*, and *HLA-DQA1* were higher expressed in *HLA-DRB1* SE-positive individuals, whereas *HLA-DQB1* was lower expressed in *HLA-DRB1* SE-positive individuals, in comparison to *HLA-DRB1* SE-negative individuals. We further confirmed the expression differences of *HLA-DQA* and *HLA-DQB* depending on *HLA-DRB1* alleles by qPCR (**Figures S1–S3**). We identified no significantly differentially expressed genes outside the MHC region in PBMCs of *HLA-DRB1* SE-positive *versus* SE-negative healthy individuals with an adjusted *P*-value < 0.05 and a fold change (log2) > 1 (**Table S1**).

To reduce heterogeneity from the effect of the second allele in the *HLA-DRB1* SE-positive group, we performed differential gene expression analysis on PBMC samples with only HLA-DRB1*03:01 as second allele. We found that the five MHC Class II genes (*HLA-DRB4*, *HLA-DQA2*, *HLA-DRB1*, *HLA-DQA1*, and *HLA-DQB1*) were differentially expressed in PBMCs of *HLA-DRB1* SE-positive *versus* SE-negative individuals carrying one HLA-DRB1*03:01 allele [adjusted *P*-value < 0.05 and a fold change (log2) > 1 (**Table S2** and **Figure 2B**)].

As the *HLA-DRB1* sequence appeared to be absent in the MHC reference haplotype APD (HLA-DRB1*13:01), we also performed differential gene expression analysis on PBMC samples of individuals with the alleles HLA-DRB1*03:01/*04:01 *versus* *03:01/*15:01. Four MHC Class II genes (*HLA-DRB5*, *HLA-DRB4*, *HLA-DQA2*, and *HLA-DQB1*) were differentially expressed in PBMCs of healthy individuals carrying HLA-DRB1*03:01/*04:01 compared to *03:01/*15:01 [adjusted *P*-value < 0.05 and a fold change (log2) > 1 (**Table S3**)]. In addition, there is a trend towards *HLA-DRB1* being higher expressed in PBMCs of individuals carrying HLA-DRB1*03:01/*04:01 compared to HLA-DRB1*03:01/*15:01, although no statistically significant difference was observed [adjusted *P*-value of 0.068 and fold change (log2) of 0.904 (**Figure 2C**)].

### Differentially Expressed Genes in CD4+ and CD8+ T Cells of *HLA-DRB1* SE-Positive *Versus* SE-Negative Healthy Individuals

**Figure 3:**
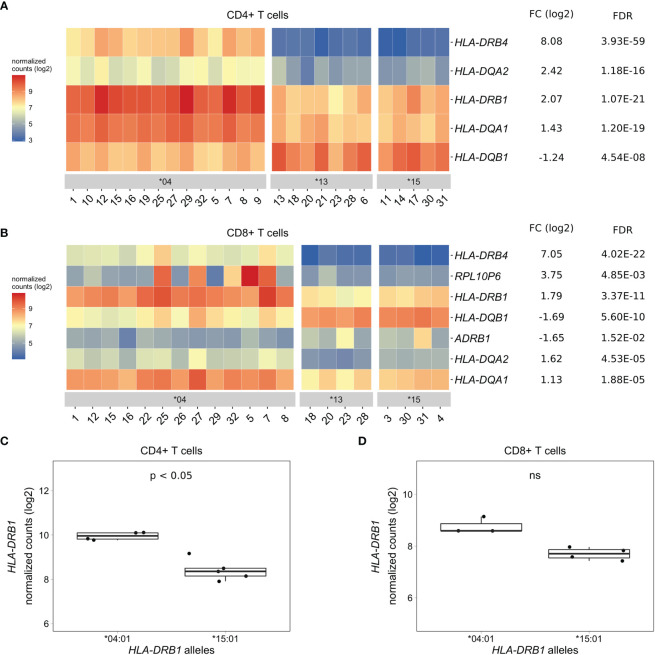
Differentially expressed genes in CD4+ and CD8+ T cells of *HLA-DRB1* SE-positive *versus* SE-negative healthy individuals. **(A)** Heat map of differentially expressed genes (log transformed normalized gene counts) in CD4+ T cells of *HLA-DRB1* SE-positive [*04 (*n* = 14)] *versus* SE-negative [*03:01/*13:01 and *03:01/*15:01 (*n* = 12)] individuals. **(B)** Heatmap of differentially expressed genes (log transformed normalized gene counts) in CD8+ T cells of *HLA-DRB1* SE-positive [*04 (*n* = 13)] *versus* SE-negative [*03:01/*13:01 and *03:01/*15:01 (*n* = 8)] individuals. In both heatmaps, genes shown in red have higher gene counts and those shown in blue have lower gene counts. **(C)** *HLA-DRB1* gene expression in CD4+ T cells of *HLA-DRB1* SE-positive [*03:01/*04:01 (*n* = 4)] and SE-negative [*03:01/*15:01 (*n* = 5)] individuals. **(D)** *HLA-DRB1* gene expression in CD8+ T cells of *HLA-DRB1* SE-positive [*03:01/*04:01 (*n* = 3)] and SE-negative [*03:01/*15:01 (*n* = 4)] individuals. FC, fold change; ns, non-significant adjusted *P*-value (FDR).

As PBMCs are a heterogeneous mixture of immune cell types, we isolated CD4+ T cells, CD8+ T cells, and CD14+ monocytes from the same healthy individuals *via* positive selection using microbeads, sequenced RNA samples that passed quality control and performed differential gene expression analyses. We found five MHC Class II genes (*HLA-DRB4*, *HLA-DQA2*, *HLA-DRB1*, *HLA-DQA1*, and *HLA-DQB1*) that were differentially expressed to a great degree in CD4+ T cells of *HLA-DRB1* SE-positive *versus* SE-negative individuals (**Figures 3A, C**, and **Tables S4–S6**). Again, we identified no differentially expressed genes outside the MHC region in CD4+ T cells of *HLA-DRB1* SE-positive *versus* SE-negative individuals (adjusted *P*-value < 0.05 and a fold change (log2) > 1 (**Tables S4–S6**)]. In CD8+ T cells, we found five MHC Class II genes (*HLA-DRB4*, *HLA-DRB1*, *HLA-DQB1, HLA-DQA2*, and *HLA-DQA1*) and two genes outside the MHC region (*RPL10P6* at chromosome 2q35 and *ADRB1* at chromosome 10q25) to be differentially expressed between *HLA-DRB1* SE-positive and SE-negative individuals (**Figure 3B** and **Tables S7–S9**). The expression differences of *HLA-DQA* and *HLA-DQB* depending on *HLA-DRB1* alleles were confirmed by qPCR (**Figures S1–S3**). In addition, there is a trend towards *HLA-DRB1* being higher expressed in CD8+ T cells of individuals carrying HLA-DRB1*03:01/*04:01 compared to HLA-DRB1*03:01/*15:01, although no statistically significant difference was observed [adjusted *P*-value of 0.538 and fold change (log2) of 0.811 (**Figure 3D**)].

### Differentially Expressed Genes in CD14+ Monocytes of *HLA-DRB1* SE-Positive *Versus* SE-Negative Healthy Individuals

**Figure 4:**
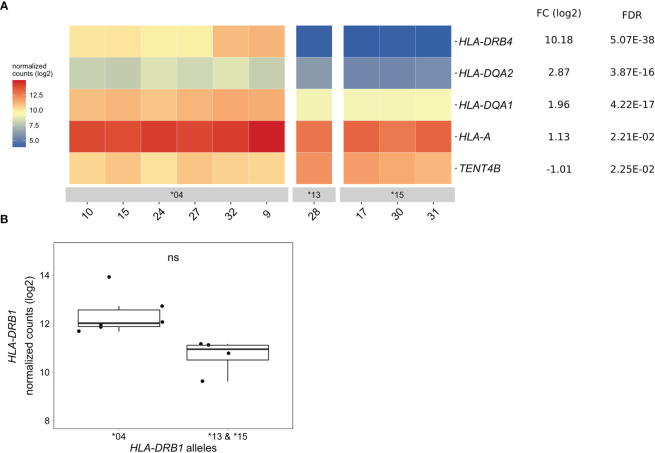
Differentially expressed genes in CD14+ monocytes of *HLA-DRB1* SE-positive *versus* SE-negative healthy individuals. **(A)** Heat map of differentially expressed genes (log transformed normalized gene counts) in CD14+ monocytes of *HLA-DRB1* SE-positive [*04 (*n* = 6)] and SE-negative [*03:01/*13:01 and *03:01/*15:01 (*n* = 4)] individuals. Genes shown in red have higher gene counts and those shown in blue have lower gene counts for the contrast SE+ *vs.* SE-. **(B)** *HLA-DRB1* gene expression in CD14+ monocytes of *HLA-DRB1* SE-positive [*04 (*n* = 6)] and SE-negative [*03:01/*13:01 and *03:01/*15:01 (*n* = 4)] individuals. FC, fold change; ns, non-significant adjusted *P*-value (FDR).

In CD14+ monocytes, we identified five genes that were differentially expressed between *HLA-DRB1* SE-positive and SE-negative healthy individuals [adjusted *P*-value < 0.05 and a fold change (log2) > 1 (**Figure 4A** and **Table S10**)]. Of these five differentially expressed genes, three were MHC Class II genes (*HLA-DRB4*, *HLA-DQA2*, and *HLA-DQA1*), one was an MHC Class I gene (*HLA-A*), and one was a non-MHC gene (*TENT4B* at chromosome 16q12). *HLA-DRB4*, *HLA-DQA2*, *HLA-DQA1*, and *HLA-A* were higher expressed in *HLA-DRB1* SE-positive individuals, whereas *TENT4B* was lower expressed in *HLA-DRB1* SE-positive individuals, in comparison to *HLA-DRB1* SE-negative individuals. In addition, there is a trend towards *HLA-DRB1* being higher expressed in CD14+ monocytes of individuals carrying HLA-DRB1*03:01/*04:01 compared to HLA-DRB1*03:01/*15:01 [adjusted *P*-value of 0.122 and fold change (log2) of 1.658 (**Figure 4B**)]. The expression differences of *HLA-DQA* and *HLA-DQB* depending on *HLA-DRB1* alleles were confirmed by qPCR (**Figures S1–S3**).

### *HLA-DRB1* Is Differentially Expressed in Whole Blood Samples of Healthy Individuals Carrying Different*HLA-DRB1* Alleles

**Figure 5:**
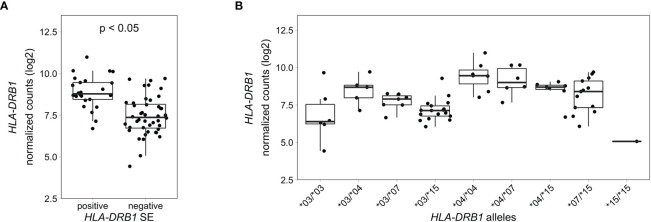
*HLA-DRB1* expression levels in whole blood samples of healthy individuals of the GTEx project. **(A)** *HLA-DRB1* gene expression in whole blood samples of *HLA-DRB1* SE-positive [*04 (*n* = 25)] and SE-negative [*03, *07, and *15 (*n* = 44)] individuals. **(B)** *HLA-DRB1* gene expression in whole blood samples of individuals carrying different combinations of *HLA-DRB1* alleles [*03/*03 (*n* = 6), *03/*04 (*n* = 5), *03/*07 (*n* = 7), *03/*15 (*n* = 17), *04/*04 (*n* = 7), *04/*07 (*n* = 6), *04/*15 (*n* = 7), *07/*15 (*n* = 13), and *15/*15 (*n* = 1)]. P, adjusted *P*-value (FDR).

To explore if this difference in *HLA-DRB1* gene expression could also be seen in whole blood samples of healthy individuals, we used RNA-seq data from 439 whole blood samples from the GTEx project (14). Classical HLA alleles were imputed from RNA-seq data and samples with HLA-DRB1*03, *04, *07 and *15 alleles were selected for mapping reads to the MHC region to avoid alignment biases. Our data show that *HLA-DRB1* is significantly higher expressed in *HLA-DRB1* SE-positive individuals (carrying at least one HLA-DRB1*04 allele) compared to *HLA-DRB1* SE-negative individuals [carrying no HLA-DRB1*04 alleles (**Figure 5A**)]. In addition, we examined the expression of *HLA-DRB1* in individuals carrying different *HLA-DRB1* alleles and determined that the expression of *HLA-DRB1* is strongly dependent on the presence of different *HLA-DRB1* alleles (**Figure 5B**). Within whole blood samples from the GTEx project, there is a trend towards higher expression levels of *HLA-DRB1* in HLA-DRB1*04 and *07 alleles compared to HLA-DRB1*03 and *15 alleles.

### *HLA-DRB1* Is Differentially Expressed in Whole Blood Samples of RA Patients Carrying Different *HLA-DRB1* Alleles

**Figure 6:**
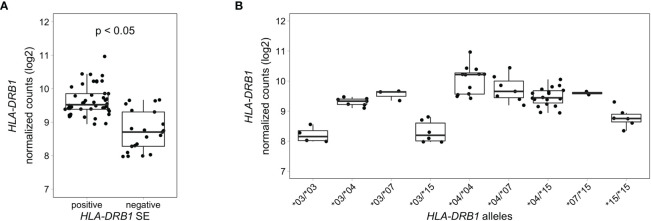
*HLA-DRB1* expression levels in whole blood samples of RA patients. **(A)** *HLA-DRB1* gene expression in whole blood samples of *HLA-DRB1* SE-positive [*04 (*n* = 44)] and SE-negative [*03, *07, and *15 (*n* = 21)] RA patients. **(B)** *HLA-DRB1* gene expression in whole blood samples of RA patients carrying different combinations of *HLA-DRB1* alleles [*03/*03 (*n* = 4), *03/*04 (*n* = 7), *03/*07 (*n* = 3), *03/*15 (*n* = 6), *04/*04 (*n* = 12), *04/*07 (*n* = 7), *04/*15 (*n* = 18), *07/*15 (*n* = 2), and *15/*15 (*n* = 6)]. P, adjusted *P*-value (FDR).

To analyze *HLA-DRB1* expression levels in RA patients carrying different *HLA-DRB1* alleles, we used RNA-seq data from 158 whole blood samples from the EIRA/RECOMBINE project. Classical HLA alleles were imputed from RNA-seq data and samples with HLA-DRB1*03, *04, *07 and *15 alleles were selected for mapping reads to the MHC region. Also in these samples, *HLA-DRB1* is significantly higher expressed in *HLA-DRB1* SE-positive RA patients (carrying at least one HLA-DRB1*04 allele) compared to *HLA-DRB1* SE-negative RA patients (carrying no HLA-DRB1*04 alleles) (**Figure 6A**). In addition, we examined the expression of *HLA-DRB1* in RA patients carrying different *HLA-DRB1* alleles and showed that the expression of *HLA-DRB1* is strongly associated with *HLA-DRB1* alleles (**Figure 6B**). In whole blood samples of RA patients, there is a trend towards higher expression levels of *HLA-DRB1* in HLA-DRB1*04 and *07 alleles compared to HLA-DRB1*03 and *15 alleles.

## Discussion

The major finding of our study is the identification of relatively high differences in gene expression levels for several MHC Class II genes in immune cells of healthy individuals depending on MHC haplotypes, which are known as genetic risk factors for autoimmune diseases. More specifically, we found that the gene for the HLA-DR beta chain is expressed higher in several types of immune cells with the RA-associated haplotype in comparison to RA-irrelevant haplotypes. By pre-selection of individuals with specific haplotypes in our study, we were able to decrease the level of heterogeneity and by accurate and haplotype-specific alignment to different MHC reference sequences, we were able to reliably identify expression levels of these genes. Our results provide for the first time evidence for differential expression of several MHC Class II genes in whole blood, PBMCs, CD4+ T cells, CD8+ T cells, and CD14+ monocytes of individuals with genetic predisposition to an autoimmune disease, i.e. RA.

The MHC is an extremely polymorphic region and quantification of expression of various allelic forms causes major issues for standard mapping methods and for studying expression of these genes. Using the standard human transcriptome reference, most of the sequencing reads will misalign to the MHC Class II locus. This could be recognized by for example *HLA-DRB5* expression within individuals carrying HLA-DRB1*04 alleles, where *HLA-DRB5* is not present (**Figures 1** and **S4**). The sequencing reads should align to *HLA-DRB4* but instead align to the most similar gene on the standard human transcriptome reference which is *HLA-DRB5*. In addition, the expression of other MHC class II genes, including *HLA-DRB1*, are also influenced by this (**Figure S4**). Here, we used the AltHapAlignR pipeline (8) for a more accurate and reliable expression analysis within the MHC region, which is especially important for the MHC Class II locus. It employs the eight available MHC reference haplotypes (APD, COX, DBB, MANN, MCF, PGF, QBL, and SSTO (24)) to generate less biased estimates of gene expression from this locus. However, the number of MHC reference haplotypes is still limited and obviously does not cover all patterns of MHC variations in the human genome. The standard MHC reference haplotype is PGF (DRB1*15:01:01-DQA1*01:02:01-DQB1*06:02). The MHC reference haplotypes COX and QBL contain the HLA-DRB1*03:01:01 allele (DRB1*03:01:01-DQA1*05:01:01-DQB1*02:01:01), DBB and MANN contain the HLA-DRB1*07:01:01 allele (DRB1*07:01:01-DQA1*02:01-DQB1*03:03:02 or DQB1*02:02, respectively), and SSTO contains the HLA-DRB1*04:03:01 allele (DRB1*04:03:01-DQA1*03:01:01-DQB1*03:05:01). In addition, *HLA-DRB1* is technically not present on the current versions of the MHC reference haplotypes APD (HLA-DRB1*13:01) and MCF [HLA-DRB1*04:01 (GRCh38.p12)]. Therefore, our findings showing that *HLA-DRB1* is lower expressed in HLA-DRB1*13:01 individuals compared to HLA-DRB1*04:01 and *15:01 are inconclusive. Furthermore, in our study the HLA-DRB1*04:01 samples are mapping to the closest matched MHC reference haplotype SSTO (HLA-DRB1*04:03:01), which could potentially decrease efficiency of alignment in the MHC Class II locus towards a lower number of mapped sequencing reads. In this case, the detected level of *HLA-DRB1* expression for individuals with HLA-DRB1*04:01 will be underestimated. The expression of *HLA-DRB4* gene, which is present at the HLA-DRB1*04 haplotype and absent at HLA-DRB1*03, *13, *15 haplotypes, was expected to be highly different between individuals carrying *HLA-DRB1* SE-positive and SE-negative alleles. Even though there are several possible issues with the MHC reference haplotypes, our data shows that the expression of *HLA-DRB* and *HLA-DQ* genes is different between individuals carrying HLA-DRB1*04:01 and *15:01 alleles in different cell types. Moreover, we found that *HLA-DRB1* tended to be higher expressed in HLA-DRB1*04 and *07 alleles compared to HLA-DRB1*03 and *15 alleles in both healthy individuals from the GTEx project and RA patients from EIRA/RECOMBINE study. Importantly, we studied the overall expression of *HLA-DRB1* and did not consider possible transcript heterogeneity due to alternative splicing that may be the source of the difference in expression.

To confirm the results of RNA-seq data, we performed qPCR analysis on the same PBMC and cell subset RNA samples. No primers could be designed to measure expression of the different forms of *HLA-DQA* (*HLA-DQA1* and *HLA-DQA2*) and *HLA-DQB* (*HLA-DQB1* and *HLA-DQB2*) due to high degree of gene homology. Therefore, we measured overall expression of *HLA-DQA* and *HLA-DQB*, and could confirm differences in expression by qPCR (**Figures S1, S2**). In addition, no differences in expression of *HLA-DRA* could be detected by qPCR (**Figure S3**), which is also concordant with our RNA-seq data. No primers can be designed for simultaneous robust measure of expression of *HLA-DRB* in samples with different *HLA-DRB1* alleles because of the high number of variations between the different forms of *HLA-DRB1*, *HLA-DRB3*, *HLA-DRB4* and *HLA-DRB5*. Therefore the expression of *HLA-DRB* was not determined by qPCR in our study.

Using RNA-seq, we found *HLA-DRB1* and other MHC Class II genes to be differentially expressed in CD4+ T cells of healthy individuals carrying *HLA-DRB1* SE-positive *versus* SE-negative alleles. Although there are CD4+ T-cell subsets expressing HLA-DR (25–27), by using xCell (22), a tool that performs cell type enrichment analysis from RNA-seq data, we noticed that the suspensions of CD4+ T cells in our experiment are slightly contaminated with monocytes (**Figure S5**). Monocytes express *HLA-DRB1* at a significantly higher level than CD4+ T cells and therefore we cannot totally exclude that the observed differences in the CD4+ T-cell subset are caused by the monocyte contamination.

HLA-DR is present on the surface of cells as heterodimers consisting of an alpha chain (HLA-DRA) and a beta chain (HLA-DRB1 together with either HLA-DRB3, HLA-DRB4 or HLA-DRB5, depending on the haplotype). Increased expression of HLA-DR is considered to be an activation marker on different cell types. Therefore, we correlated *HLA-DRB1* expression levels with the expression of other activation markers of different cell types (*CD25*, *CD38*, *CD69*, *CD86*, *CD40*, and *CD63*). We found no correlation with any of these activation markers (**Figures S6–S9**), suggesting that the higher expression of *HLA-DRB1* in samples with HLA-DRB1*04 alleles in our study cannot be due to uncontrolled cell activation.

Our study shows that HLA-DR is not only expressed in professional antigen presenting cells, such as CD14+ monocytes, but also in CD4+ and CD8+ T cells. Molecular mechanisms of differences in gene expression levels for *HLA-DRB1* in these immune cells of healthy individuals and RA patients depending on MHC haplotypes are not addressed in this study. Several studies have suggested that the levels of *HLA-DRB1* might be regulated by DNA methylation (28–30), which might be distinctive for different haplotypes. They show that hypomethylation of the *HLA-DRB1* promoter, which leads to higher expression of *HLA-DRB1*, is involved in the pathogenesis of for example multiple sclerosis. In addition, there is evidence that the expression of *HLA-DRB1* is regulated by super enhancers located in the MHC region between the genes *HLA-DRB1* and *HLA-DQA1* (31–33). Histone modifications in these enhancers are associated to differential 3D chromatin conformation and gene expression of HLA genes, and more importantly, there seem to be differences in individuals carrying different MHC haplotypes (34). The super enhancers were found to regulate HLA-DR and HLA-DQ protein levels as well (32, 33). This suggests that not only differences at *HLA-DRB* and *HLA-DQ* gene expression level can be observed in individuals with different MHC haplotypes, but also at protein level. However, studies identifying functional consequences of higher gene expression levels of *HLA-DRB1* in HLA-DRB1*04 individuals are still pending.

Using the available MHC reference haplotypes, we were able to identify by RNA-seq that *HLA-DRB* and *HLA-DQ* genes are differentially expressed in whole blood samples, PBMCs, CD4+ T cells, CD8+ T cells, and CD14+ monocytes of *HLA-DRB1* SE-positive *versus* SE-negative healthy individuals. In addition, we identified that *HLA-DRB1* is also differentially expressed in whole blood samples of *HLA-DRB1* SE-positive *versus* SE-negative RA patients. While MHC Class II genes consistently demonstrate differential expression between *HLA-DRB1* SE-positive and SE-negative healthy individuals, we found little difference for expression of non-MHC genes. In conclusion, our study shows that not only structural differences but also differences in expression of MHC Class II molecules in different immune cells may explain the relationships between immunity and autoimmune disease and presence of certain MHC Class II alleles in an individual.

## Data Availability Statement

All sequencing data generated in this study are available at NCBI Gene Expression Omnibus accession number GSE163605.

## Ethics Statement

The studies involving human participants were reviewed and approved by Regional Ethical Review Board in Uppsala. The patients/participants provided their written informed consent to participate in this study.

## Author Contributions

MH: Conceptualization, Methodology, Software, Validation, Formal analysis, Investigation, Data Curation, Writing – Original Draft, Visualization. EH: Investigation, Writing – Review & Editing. LR: Resources, Writing – Review & Editing. LK: Conceptualization, Writing – Review & Editing. VM: Writing – Review & Editing. LP: Conceptualization, Methodology, Data Curation, Writing – Review & Editing, Supervision, Project administration, Funding acquisition. All authors contributed to the article and approved the submitted version.

## Funding

This work was supported by the Swedish Research Council [2015-3006 (LP), 2018-2399 (LR) and 2018-2884 (LP)], the Swedish Rheumatism Association (LR), King Gustav V’s 80-year Foundation (LR) and Börje Dahlin Foundation (LP). The funders had no role in study design, data collection and analysis, decision to publish, or preparation of the manuscript.

## Conflict of Interest

The authors declare that the research was conducted in the absence of any commercial or financial relationships that could be construed as a potential conflict of interest.

## Publisher’s Note

All claims expressed in this article are solely those of the authors and do not necessarily represent those of their affiliated organizations, or those of the publisher, the editors and the reviewers. Any product that may be evaluated in this article, or claim that may be made by its manufacturer, is not guaranteed or endorsed by the publisher.
