# Supplementary material for: Haplotype-Specific Expression Analysis of MHC Class II Genes in Healthy Individuals and Rheumatoid Arthritis Patients
Source: Front Immunol. 2021 Aug 17;12:707217. doi: 10.3389/fimmu.2021.707217 (PMC8416041; doi:10.3389/fimmu.2021.707217)
Supplement: Supplementary file 1 [file DataSheet_1.pdf]

Supplementary Material

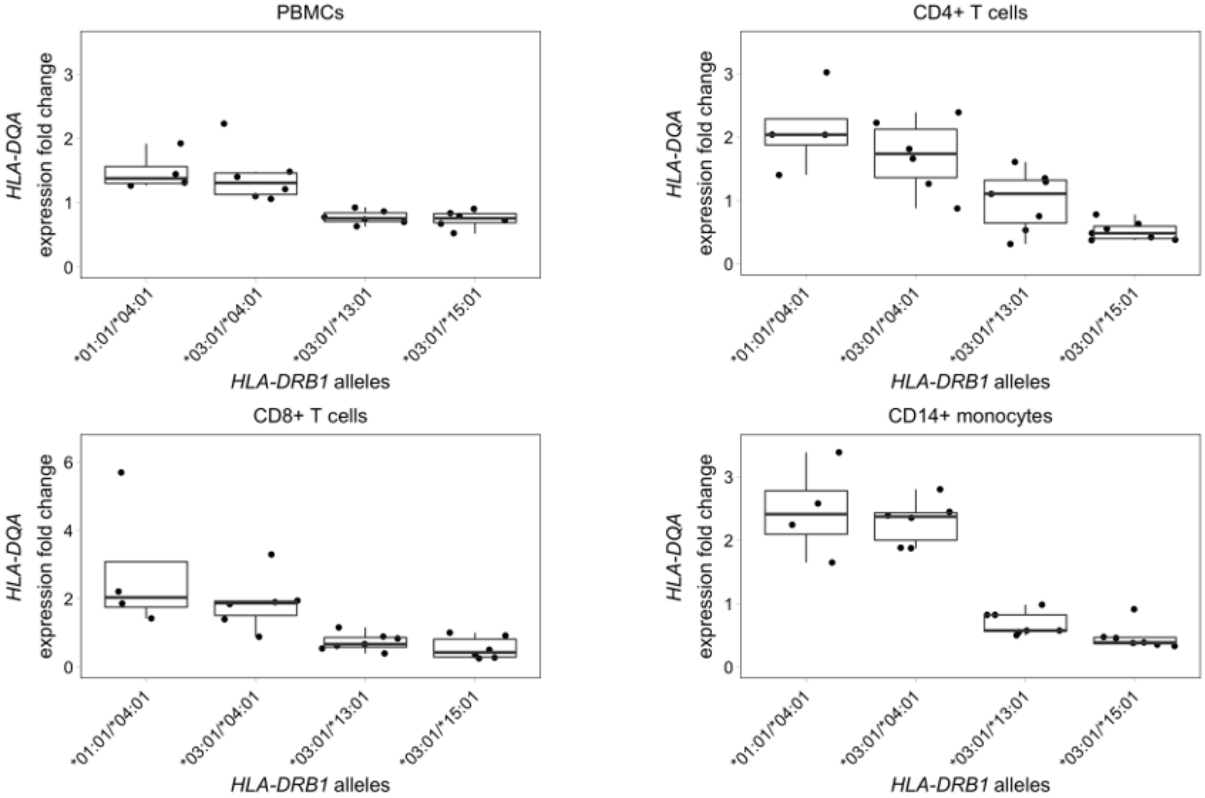

Figure S1. *HLA-DQA* expression levels in different cell types of healthy individuals carrying different *HLA-DRB1* alleles. *HLA-DQA* expression in PBMCs, CD4+ and CD8+ T cells, and CD14+ monocytes of individuals carrying *HLA-DRB1**01:01/*04:01, *HLA-DRB1**03:01/*04:01, *HLA-DRB1**03:01/13:01, and *HLA-DRB1**03:01/*15:01 alleles obtained by quantitative real-time PCR. The endogenous controls *ACTIN*, *UBE2D2* and *ZNF592* were used to normalize the expression levels of *HLA-DQA*.

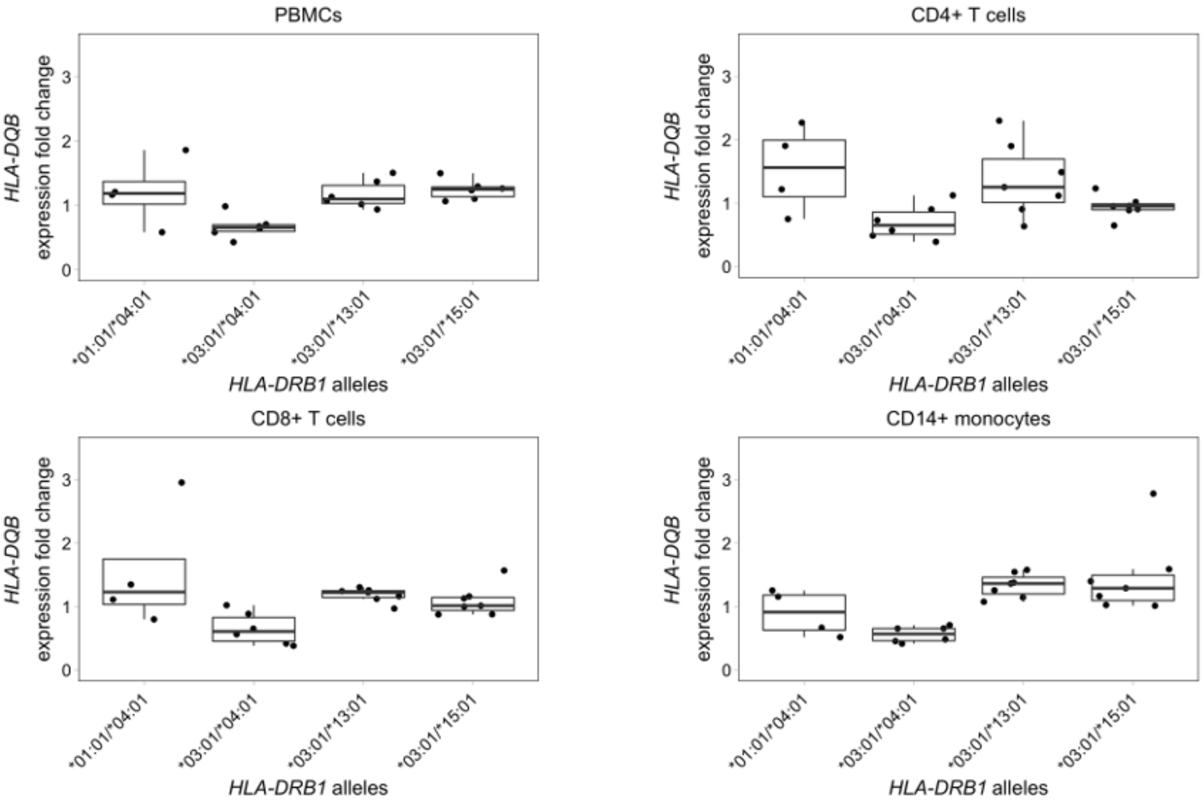

Figure S2. *HLA-DQB* expression levels in different cell types of healthy individuals carrying different *HLA-DRB1* alleles. *HLA-DQB* expression in PBMCs, CD4+ and CD8+ T cells, and CD14+ monocytes of individuals carrying *HLA-DRB1**01:01/*04:01, *HLA-DRB1**03:01/*04:01, *HLA-DRB1**03:01/13:01, and *HLA-DRB1**03:01/*15:01 alleles obtained by quantitative real-time PCR. The endogenous controls *ACTIN*, *UBE2D2* and *ZNF592* were used to normalize the expression levels of *HLA-DQB*.

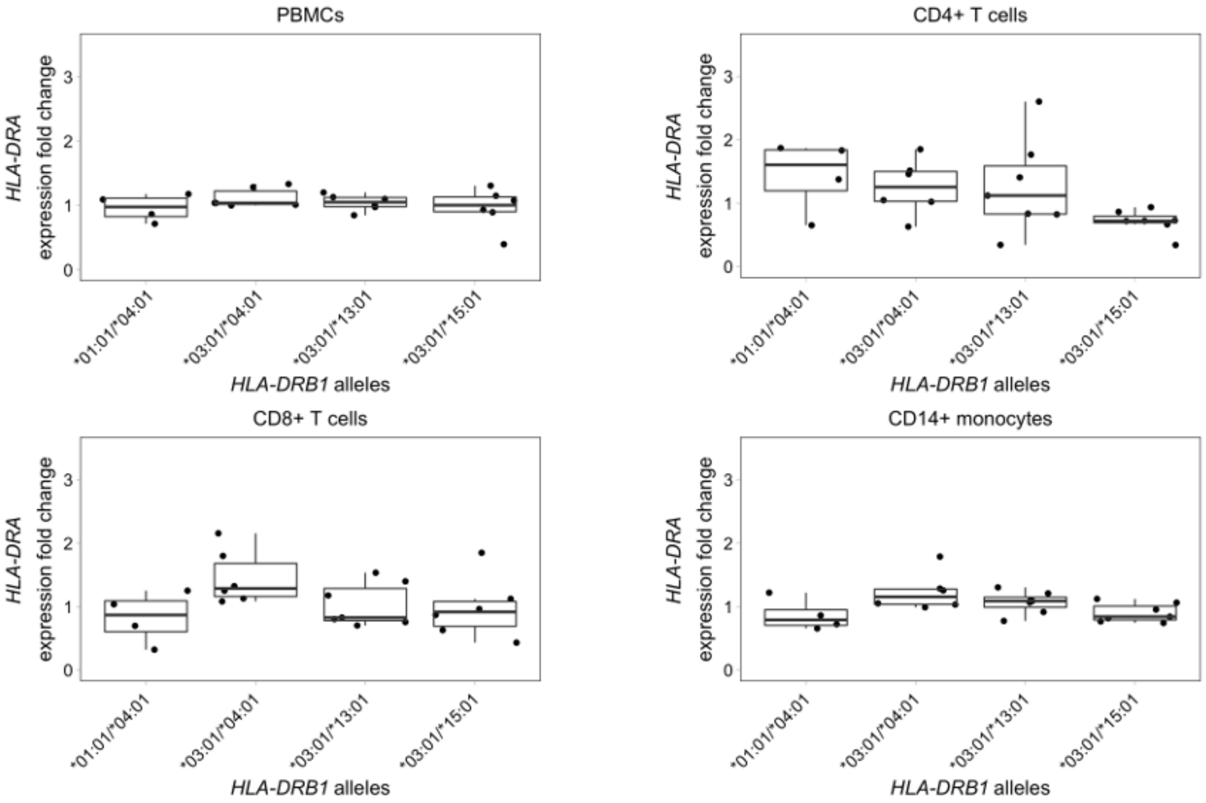

Figure S3. *HLA-DRA* expression levels in different cell types of healthy individuals carrying different *HLA-DRB1* alleles. *HLA-DRA* expression in PBMCs, CD4+ and CD8+ T cells, and CD14+ monocytes of individuals carrying *HLA-DRB1**01:01/*04:01, *HLA-DRB1**03:01/*04:01, *HLA-DRB1**03:01/13:01, and *HLA-DRB1**03:01/*15:01 alleles obtained by quantitative real-time PCR. The endogenous controls *ACTIN*, *UBE2D2* and *ZNF592* were used to normalize the expression levels of *HLA-DRA*.

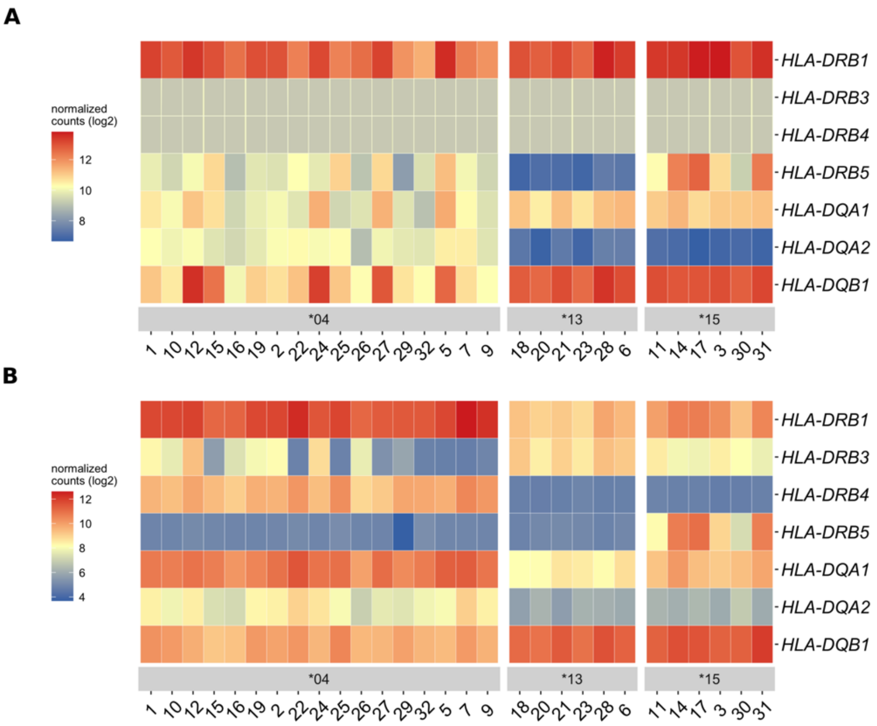

Figure S4. MHC Class II gene expression in PBMCs of *HLA-DRB1* SE-positive versus SE-negative healthy individuals using different pipelines. A) Log transformed normalized gene counts of MHC Class II genes in PBMCs of *HLA-DRB1* SE-positive [*04 (*n* = 17)] versus SE-negative [*03:01/*13:01 and *03:01/*15:01 (*n* = 12)] individuals obtained using the standard MHC reference haplotype PGF. **B)** Log transformed normalized gene counts of MHC Class II genes in PBMCs of the same *HLA-DRB1* SE-positive versus SE-negative individuals obtained using all currently available MHC reference haplotypes (APD, COX, DBB, MANN, MCF, PGF, QBL, and SSTO). Genes shown in red have higher gene counts and those shown in blue have lower gene counts.

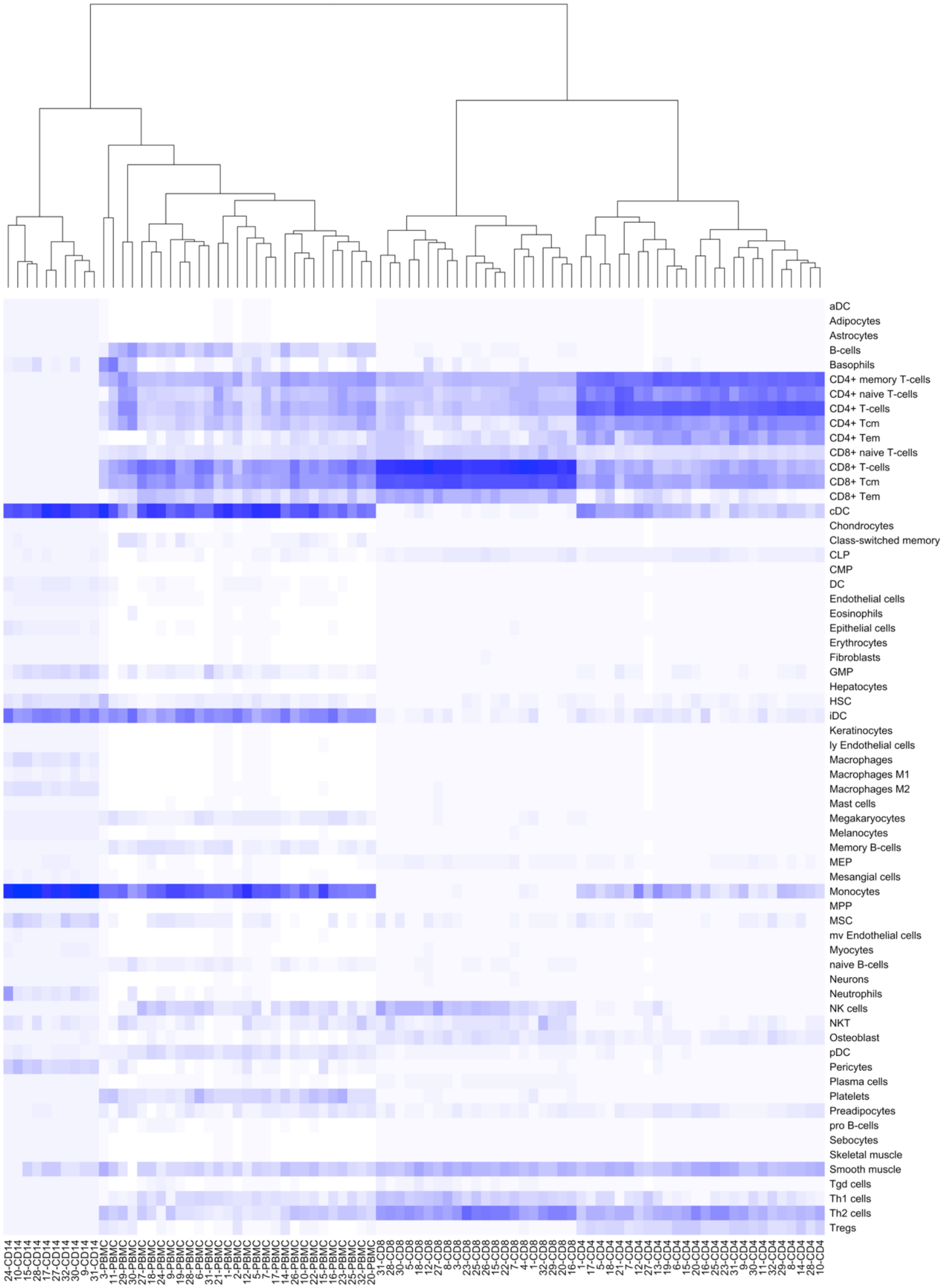

Figure S5. Cell type enrichment analysis in the different isolated cell subsets. Clustering heatmap showing cellular heterogeneity in PBMC, CD4+ T-cell, CD8+ T-cell, and CD14+ monocyte subsets. The xCell tool [13] was used to identify cellular heterogeneity in the different isolated cell subsets from gene expression data. Intensity of the blue color indicates relative cell type enrichment.

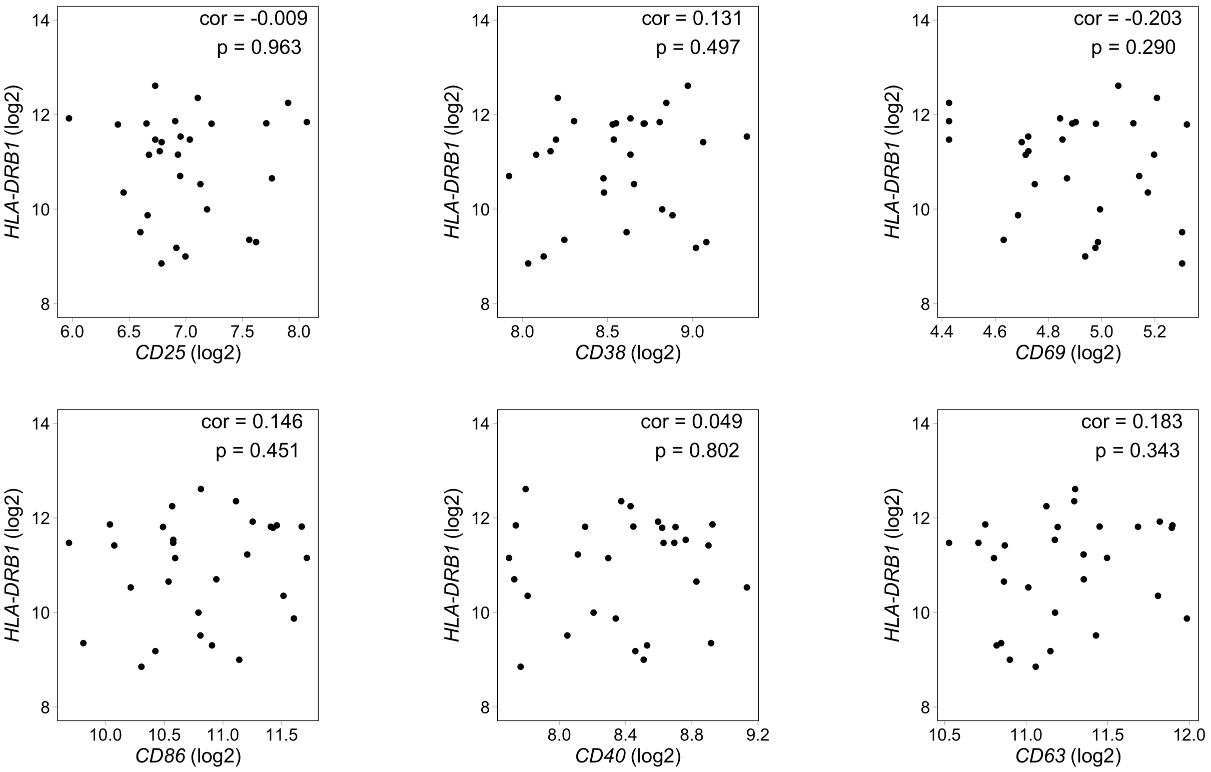

Figure S6. Correlation of gene expression levels of *HLA-DRB1* and other activation markers in PBMCs of healthy individuals. Expression levels of *HLA-DRB1*, *CD25*, *CD38*, *CD69*, *CD86*, *CD40*, and *CD63* are shown as log transformed normalized gene counts. In each subplot, cor is Pearson correlation.

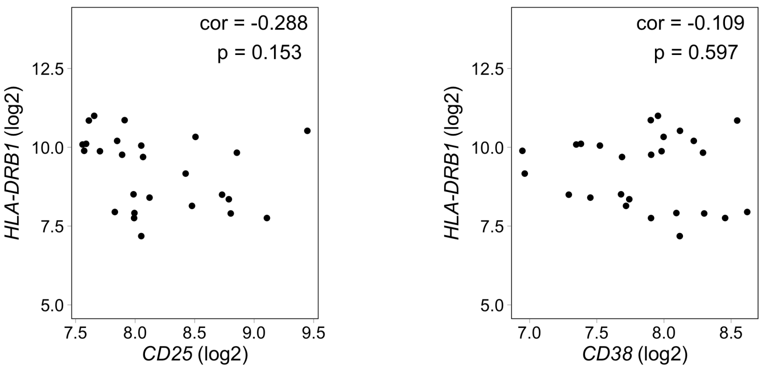

Figure S7. Correlation of gene expression levels of *HLA-DRB1* and other activation markers in CD4+ T cells of healthy individuals. Expression levels of *HLA-DRB1*, *CD25*, and *CD38* are shown as log transformed normalized gene counts. In each subplot, cor is Pearson correlation.

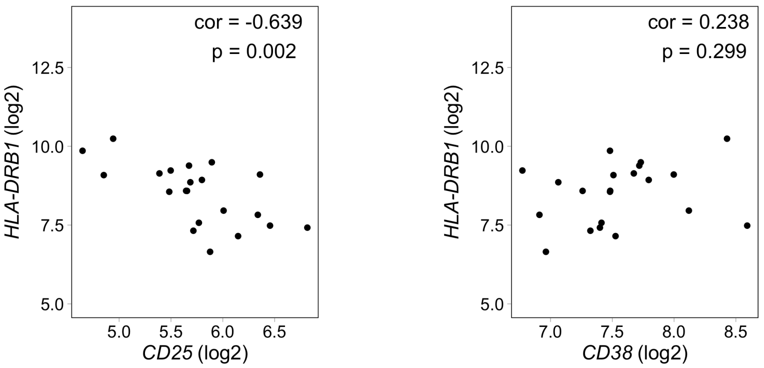

Figure S8. Correlation of gene expression levels of *HLA-DRB1* and other activation markers in CD8+ T cells of healthy individuals. Expression levels of *HLA-DRB1*, *CD25*, and *CD38* are shown as log transformed normalized gene counts. In each subplot, cor is Pearson correlation.

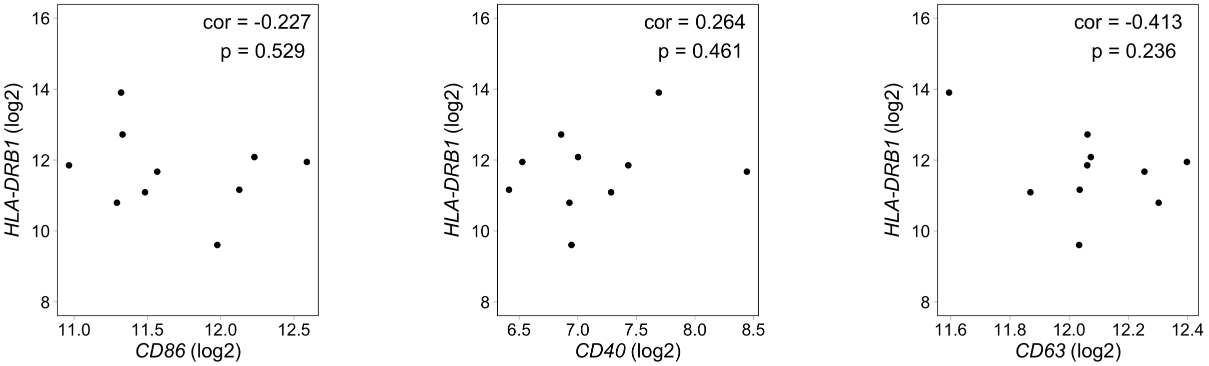

Figure S9. Correlation of gene expression levels of *HLA-DRB1* and other activation markers in CD14+ monocytes of healthy individuals. Expression levels of *HLA-DRB1*, *CD86*, *CD40*, and *CD63* are shown as log transformed normalized gene counts. In each subplot, cor is Pearson correlation.
